# Evaluation of the Safety and Immunogenicity of a Peste Des Petits Ruminants (PPR), Sheep and Goat Pox (SGP) and Contagious Caprine Pleuropneumonia (CCPP) Multivalent Vaccine (JOVAC-Combo^®^) Under Field Conditions in Somalia

**DOI:** 10.3390/v18091014

**Published:** 2026-09-14

**Authors:** Ahmad M. Almajali, Hazar Shawash, Hassan Salaita, Mana Mahapatra, Satya Parida, Stephen Wilson

**Affiliations:** 1 Department of Veterinary Clinical Sciences, Faculty of Veterinary Medicine, Jordan University of Science and Technology, Irbid 22110, Jordan; 2 World Organisation for Animal Health (WOAH), 12 Rue de Prony, 75017 Paris, France; 3 Jordan Bio-Industries Center (JOVAC), Amman 11941, Jordan; 4 The Pirbright Institute, Surrey GU24 0NF, UK; 5 Food and Agriculture Organization of the United Nations (FAO), Viale delle Terme di Caracalla, 00153 Rome, Italy; 6 Royal Veterinary College, University of London, London NW1 0TU, UK; 7 GALVmed, Pentlands Science Park, Bush Loan, Penicuik, Edinburgh EH26 0PL, UK

**Keywords:** peste des petits ruminants (PPR), sheep and goat pox (SGP), contagious caprine pleuropneumonia (CCPP), multivalent vaccine, combo vaccine, immunogenicity, safety

## Abstract

Peste des petits ruminants (PPR), sheep and goat pox (SGP), and contagious caprine pleuropneumonia (CCPP) are diseases of small ruminants and are the major constraints to livestock trading in Africa, the Middle East, and Asia. Vaccination against these diseases individually is challenging to veterinary authorities due to high cost and logistics. In this study, we evaluate the safety and immunogenicity of a multivalent vaccine (JOVAC-Combo^®^) in goats in Somalia that induced seroconversion against all three pathogens, PPR, SGP, and CCPP. A total of 120 apparently healthy, seronegative young goats (4–9 months old) from three different villages in Somalia were enrolled (90 vaccinated with JOVAC-Combo^®^ and 30 controls) and monitored for evidence of adverse effects and serological response for 90 days post-vaccination. JOVAC-Combo^®^ was found to be safe and immunogenic, with no detectable side effects. JOVAC-Combo^®^ induced humoral immunity by 14 days post-vaccination; at 30 days post-vaccination, antibody levels were high for all three pathogens, and they remained high until the end of the study (day 90). Whether immunity persists beyond the study period, and whether it translates into protection against clinical disease, remains to be established. These findings present a positive indication for the use of a cost-effective multivalent vaccine for supporting the national and regional efforts to control and reduce impacts of PPR, SGP, and CCPP infections in endemic areas.

## 1. Introduction

Peste des petits ruminants (PPR), sheep and goat pox (SGP), and contagious caprine pleuropneumonia (CCPP) are major transboundary infectious animal diseases that affect small ruminants and can have large economic impacts [1,2,3]. These impacts include large production losses, markedly increased mortality and morbidity (especially in young animals), and reduced reproductive performance [2,4,5]. These diseases impose a major economic burden not only on farmers but also on affected countries; PPR alone causes economic losses estimated at USD 1.2–1.7 billion each year, one-third of which is incurred in Africa and a quarter in South Asia [6,7,8]., while CCPP alone has an estimated yearly economic impact of around USD 507 million [9].

PPR, SGP, and CCPP in small ruminants are endemic in most African countries and in some Asian countries (especially the Middle East), causing significant economic losses for farmers and severe damage to animal health and welfare. Importantly, these diseases need to be differentially diagnosed in the field and confirmed in the laboratory. A systematic review and meta-analysis of PPR in Africa and Asia reported pooled prevalence estimates of 40.99% (95% CI: 37.20%–44.79%) in Africa and 38.43% (95% CI: 35.64%–41.22%) in Asia [10]. The seroprevalence of SGP was calculated from studies included in a systematic review with a cumulative sample size of 4352 animals, and the prevalence was found to be significantly high, ranging between 8.2% and 38.2% [7,11,12,13,14]. The pooled prevalences of CCPP worldwide were determined using a random-effects meta-analysis model. Prevalence of CCPP was 23.19% (95% CI: 11.90–34.47%) in sheep and 24.91% (95% CI: 20.99–28.84%) in goats. Overall, the regional-level pooled prevalence estimates ranged from 8.0% (95% CI: 6.91–9.09%) to 28.70% (22.02–35.38%), depending on species and world region [3].

Control of PPR, SGP, and CCPP in endemic areas depends mainly on vaccination. Small ruminant vaccination is challenged by the high cost of vaccines, vaccine implementation and the lack of logistics, especially in the field. Regular roadmap meetings of the WOAH and the Food and Agriculture Organization of the United Nations (FAO) in Africa and Asia recommended the adoption of multivalent vaccines that should reduce costs and logistics. In this study, we describe the safety and immunogenicity of a novel trivalent vaccine (JOVAC-Combo^®^) against PPR, SGP, and CCPP under field conditions in Somalia.

## 2. Materials and Methods

### 2.1. Ethical Statement

The animal study protocol was approved by the Institutional Animal Care and Use Committee (IACUC) of Jordan University of Science and Technology (Protocol No. 6-12-20, Date of Approval: 12 June 2020) and by the Jordan Bio-Industries Center (JOVAC) Ethical Review Committee. Field activities in Somalia were conducted in collaboration with the responsible Somali veterinary authorities. All animal experiments were conducted in accordance with national and international guidelines for the care and use of laboratory animals and complied with relevant ethical regulations.

### 2.2. Study Location

Three villages, namely Garash, Qoqane, and Marergabta, in Beletweyn district of Somalia, were selected for this study. The selection criteria of these villages included the density of sheep and goat populations, the incidence of PPR, SGP, and CCPP diseases, as well as the occurrence of disease outbreaks in the region. Garash and Qoqane are located near a river, making them riverine villages. On the other hand, Marergabta is a village that relies on both agriculture and animal husbandry, making it an agro-pastoral community. The three villages are located inside the Beletweyn district, which is part of the Hiran area in the Hirshabelle state of Somalia. The Somali livestock population is predicted to be 57,177,652, consisting of 7,173,988 camels, 5,319,533 cattle, 30,998,566 goats, and 13,685,565 sheep.

### 2.3. Vaccine Tested

A trivalent vaccine against peste des petits ruminants (PPR), sheep and goat pox (SGP), and contagious caprine pleuropneumonia (CCPP)—JOVAC-Combo^®^ (Batch No. COK0123, Jordan Bio-Industries Center, Amman, Jordan)—was used. The vaccine is supplied as two components: a liquid formulation containing live attenuated PPR virus (strain Nig 75/1) and live attenuated Kenyan sheep and goat pox virus (SGP, strain 0240) stabilized in a liquid stabilizer (Arecor Therapeutics PLC, Cambridge, UK), and a freeze-dried live *Mycoplasma capricolum* subsp. *capripneumoniae* (Mccp) component. Immediately before vaccination, the freeze-dried Mccp component was reconstituted in the liquid PPR/SGP formulation, essentially following the manufacturer’s instructions. Each 1 mL dose contains a minimum of 10^2.5^ TCID_50_ of the live attenuated PPR virus, a minimum of 10^2.5^ TCID_50_ of the live attenuated KSGP virus and, for the CCPP component, a minimum of 10^5.5^ color-changing units (CCU) of freeze-dried Mccp. JOVAC release-certificate of analysis for Batch no COK0123 indicates that the actual potency of the vaccine for PPRV is 10^3.6^ TCID_50_; for SGV is 10^3.8^ TCID_50_ and for Mccp is 10^6.4^ CCU.

### 2.4. Animal Study

Apparently healthy goats of the Short-eared Somali goat breed were sourced from three different villages (Marergabta, Qoqane, and Garash) in Hirshabelle State, Republic of Somalia. From each village, 40 goats of either sex were enrolled, giving a total of 120 goats, and all enrolled goats were ear-tagged for identification. The goats were 4–9 months old at enrolment, had no history of previous vaccination against PPR, SGP or CCPP, and were seronegative for all three pathogens by the assays used (day 0). Inclusion required a normal demeanor and appetite with no clinical signs on veterinary examination. In each village, the 40 goats were randomly assigned to a vaccinated group (*n* = 30) or a control group (*n* = 10). Stratification according to age or sex when we did randomization was not implemented. The freshly reconstituted JOVAC-Combo^®^ vaccine was administered (1 mL subcutaneously) to either the left or the right side of the neck in the vaccinated group, while the remaining 10 goats per village were mock-vaccinated with 1 mL sterile phosphate-buffered saline (PBS) by the same subcutaneous route and on the same schedule (single dose at day 0) to serve as controls. Animals were observed twice daily, and rectal temperatures and adverse effects were recorded for at least the first 10 days by veterinary personnel; weekly check-ups were carried out until the end of the study (90 days post-vaccination). An adverse event was defined as any local reaction (swelling, abscess, or injection-site lesion) or systemic reaction (rectal temperature ≥ 40.5 °C, anorexia, depression, anaphylaxis, or death) observed within 10 days of vaccination and considered related to the vaccine (Rectal Temperatures observed for 7 days post vaccination not 10 days). Blood samples were collected at days 0 (pre-vaccination), 14, 30, 60, and 90 days post-vaccination for serum preparation. The clotted blood samples were transported to a laboratory in Somalia, where serum was separated and stored at +4 °C. Serum samples were heat-inactivated at 56 °C for 30 min prior to refrigerated shipment to JOVAC, Jordan, for further analysis. All 120 goats completed the 90-day follow-up; none was lost to follow-up, and blood samples were obtained from all animals at every scheduled time point. Field staff and laboratory personnel blinded to group allocation; serum samples were labeled with individual ear-tag identifiers.

Due to limited resources and since the vaccine at the time of investigation was not registered in Somalia, the sample size was set to 40 goats per group. We did not follow any formulas for sample size calculations as the number of goats per group is expected to be higher than the animals that we were able to allocate.

### 2.5. Laboratory Analysis

Analysis of the serum samples for the presence of antibodies against PPR virus (PPRV), sheep and goat pox virus (SGPV) and CCPP was carried out in the R&D division of JOVAC, Amman, Jordan. Samples were screened for PPRV antibodies using the IDvet ID Screen^®^ PPR Competition ELISA (Innovative Diagnostic, Lyon, France) following the manufacturer’s instructions; samples were tested in duplicate, kit positive and negative controls were included on each plate, and borderline samples were re-tested (cut off; positive S/N% ≤ 50 is considered positive). Antibodies against CCPP were detected using a CCPP competitive ELISA kit (Idexx Laboratories, Westbrook, ME, USA) according to the manufacturer’s protocol, with the manufacturer-specified percentage-inhibition (PI%) (cut-off ≥ 55 is considered positive); the assay is used according to the manufacturer’s instructions for goat sera. Antibodies against capripoxvirus (SGP) were detected using an in-house Immunoperoxidase Monolayer Assay (IPMA) performed at JOVAC, testing in duplicates and recorded as positive or negative. In-house positive and negative sera were included as controls in each IPMA run, and assay repeatability data from routine diagnostic validation are available on request. All tests used were validated for applicability in goats in house using the control sera.

### 2.6. Statistical Analysis

ELISA S/N ratios, CCPP percentage inhibition values, and IPMA results for animals in the vaccinated and control groups were analyzed using a linear mixed-effects model with fixed effects for group, time and their interaction, animal as a random effect to account for repeated measurements, and village as a random (cluster) effect to account for the nested study design. Seropositivity (binary outcome) was modeled using a generalized linear mixed model (binomial family, logit link). Model-based pairwise contrasts between groups at each time point are reported; a threshold for significance of *p* = 0.05 was used. All analyses were implemented using the Statistical Package for the Social Sciences (IPM SPSS) version 20 (Chicago, IL, USA).

## 3. Results

### 3.1. Vaccine Safety

**Figure 1:**
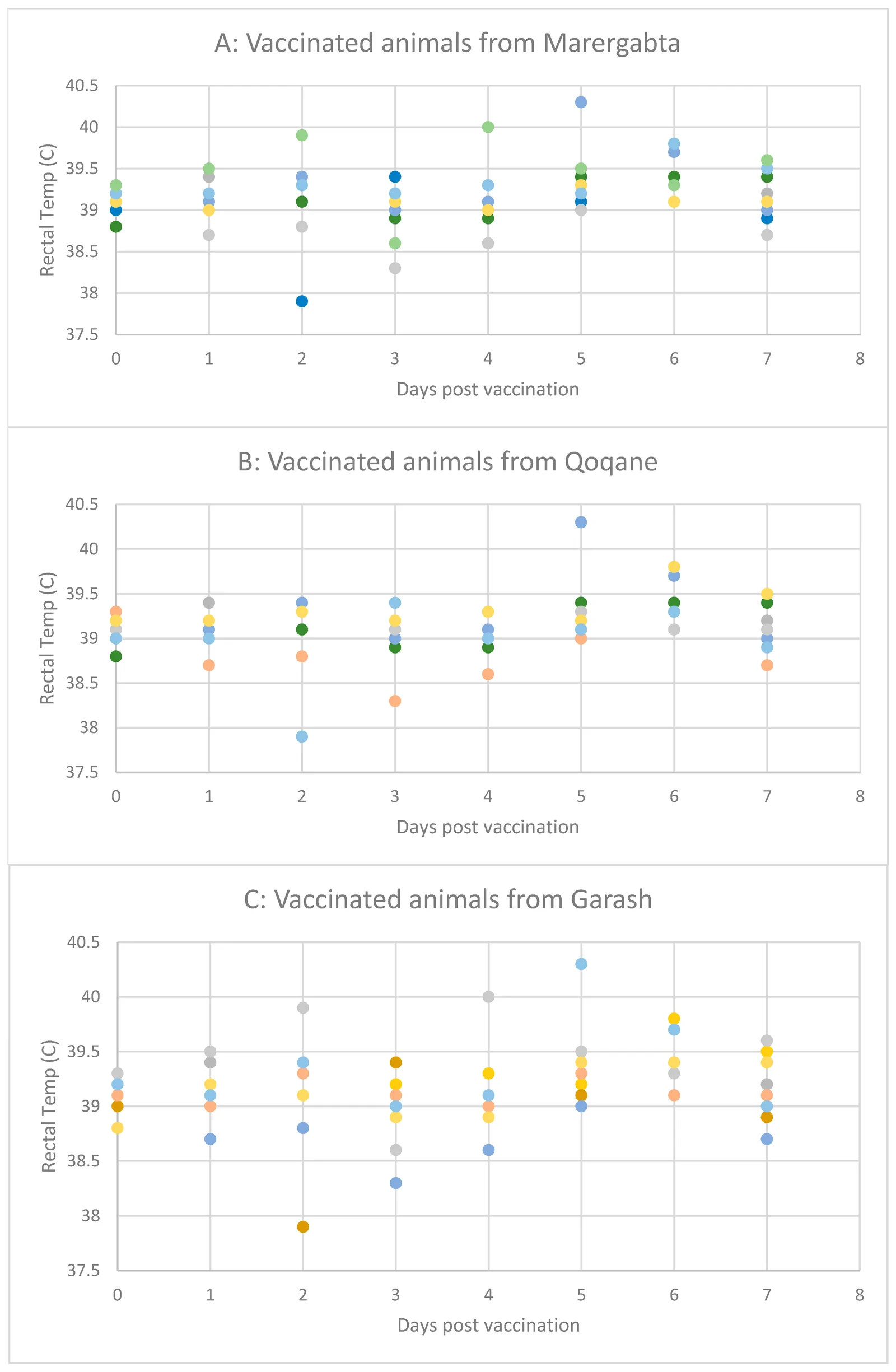
Frequency of rectal temperatures of goats vaccinated with JOVAC-Combo^®^ (vaccinated groups) and mock-vaccinated control goats. (**A**) Vaccinated animals in a herd in Marergabta; (**B**) vaccinated animals in a herd in Qoqane; (**C**) vaccinated animals in a herd in Garash. Rectal temperature recordings cover days 0–7 post-vaccination. Each color point on each day may represent more than one reading with the same value (frequency).

The three antigens used in the JOVAC-Combo vaccine, when administered individually to target species in the field, have been reported to be safe and effective. However, administration of all three antigens in a single shot warrants study to evaluate the safety and immunogenicity of the multivalent vaccine. Throughout the course of this study, goats were examined by veterinary personnel to determine whether there were any localized or systemic adverse effects that could be attributed to the administered vaccine formulation; adverse events were defined in Section 2.4. No such adverse effects were observed. There was no significant difference in body temperatures between the vaccinated and control animals (Figure 1).

### 3.2. Antibody Response

**Figure 2:**
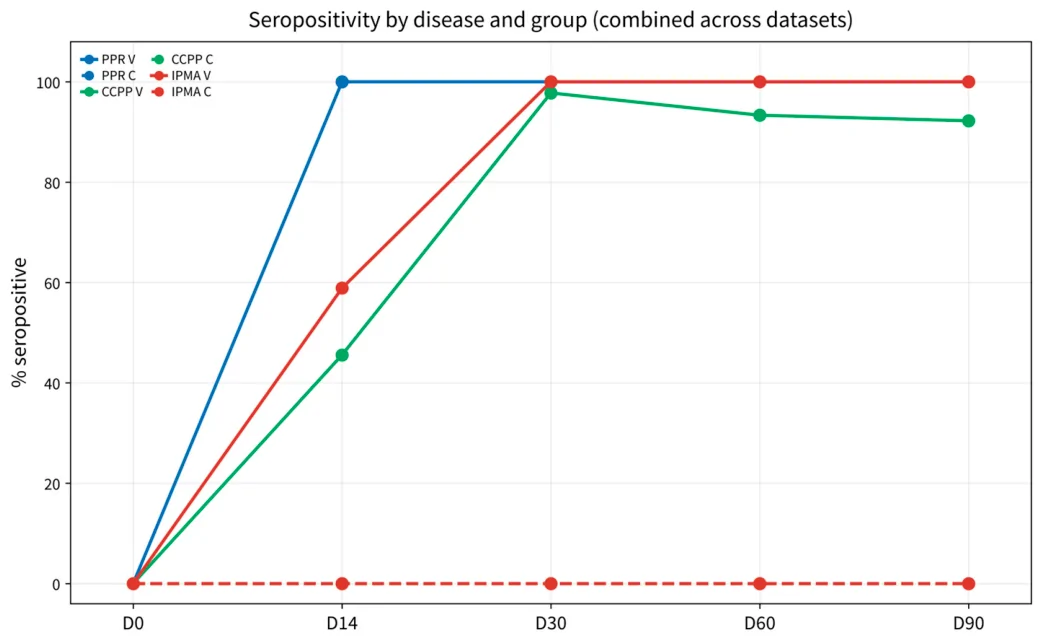
Seropositivity by disease and group; solid = vaccinated, dashed = control.

**Figure 3:**
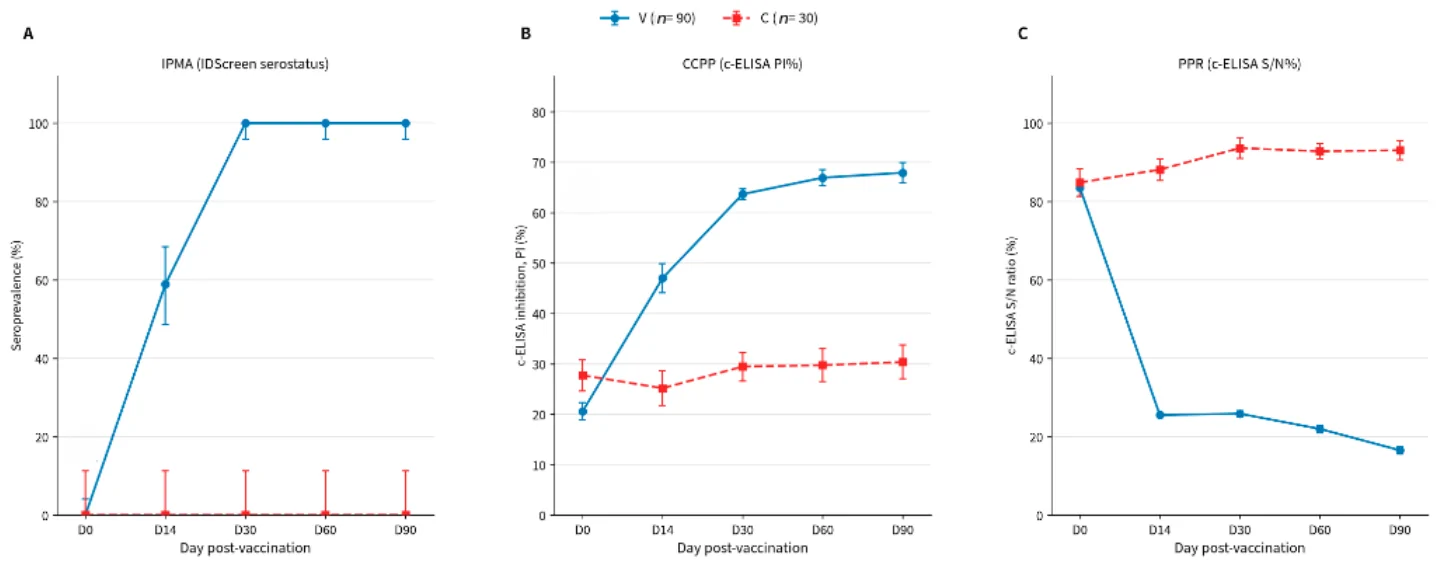
Mean with 95% CI over time. PPR S/N %, CCPP PI %, IPMA % seropositive. Solid lines = vaccinated (V); dashed = control (C). PPR *n* = 120 (planned panel size; supplied sheets contain 120 longitudinal animal records), CCPP *n* = 120, IPMA *n* = 120. Error bars denote the 95% CI (t-based interval for CCPP PI % and PPR S/N %; Wilson interval for IPMA seroprevalence). For all time points (D14, D30, D60 and D90) the *p* value < 0.001; in D0 no significant differences were detected.

A strong serological response was observed in vaccinated animals for all three vaccine components across the three study villages (Figure 2). For PPR, seropositivity increased from 0% (95% CI: 0.0–4.0%) at D0 to 100% (95% CI: 96.0–100%) by D14 and remained at this level through D90, while all control animals remained seronegative throughout the study period. Similarly, for the SGP, no vaccinated animals were seropositive at D0, whereas seropositivity reached approximately 63% (95% CI: 53–73%) at D14 and 100% (95% CI: 96.0–100%) from D30 through D90. In contrast, all control animals remained seronegative during follow-up. For the CCPP component, seropositivity rose from 0% (95% CI: 0.0–4.0%) at D0 to approximately 57% (95% CI: 46–67%) at D14, increasing further to 97% (95% CI: 91–99%) at D30 and remaining high at D60 (93%, 95% CI: 85–97%) and D90 (90%, 95% CI: 82–95%). Control animals remained seronegative throughout the study (Figure 3). Overall, these findings demonstrate rapid and sustained seroconversion following vaccination, with complete and highly consistent responses observed for PPR and SGP, while CCPP induced a strong but slightly more variable antibody response over time.

## 4. Discussion

Combination vaccines are well known for reducing distress to the vaccinees and are also likely to enhance vaccine uptake rates. Many combination vaccines are reported to be as efficacious as the individual vaccines; however, an increasing number of antigens in a vaccine could theoretically pose problems in terms of reduced immunogenicity or increased reactogenicity. To our knowledge, this is the first field evaluation of a commercial freeze-dried trivalent vaccine that combines PPR (Nig 75/1), SGP (KSGP 0240), and CCPP (Mccp) antigens (JOVAC-Combo^®^) in goats. These findings present a positive indication for the use of a multivalent vaccine for the control and eradication of PPR, SGP, and CCPP by veterinary authorities in different countries in Africa and Asia.

Several studies described the efficacy and value of a bivalent vaccine containing PPR Nig 75/1 and SGP strain KSGP 0240 [5,15,16,17]. No difference in immunity between the monovalent and bivalent vaccines was reported [15,16,18]. In our investigation, combining those two vaccine strains with *Mycoplasma capricolum* subspecies *capripneumoniae* (Mccp) did not appear to impair the immune response to any of the three components; however, formal assessment of synergy or interference would require a comparative study that includes groups receiving each component individually. In a recent study, Hurisa and colleagues vaccinated goats with four vaccine antigens, namely PPR, CCPP, SGP, and Pasteurellosis, in different combinations, either as mono-, bi-, tri-, or tetravalent [19]. They reported the vaccines to be safe and immunogenic for all antigens except SGP; even the group containing the monovalent SGP vaccine did not seroconvert. They suggested either that the SGP vaccine formulation was not efficacious or that the antibody response to the SGP vaccine was suppressed because too many antigens were administered at the same time. In this study, the trivalent vaccine was found to be safe and immunogenic. Hosamani and colleagues also demonstrated that a bivalent vaccine containing PPR and SGP antigens was safe and effective and protected animals from virulent virus challenge [17].

Multi-valent vaccines offer pragmatic and cost-effective disease-control tools for the small-scale livestock keeper, especially the most vulnerable. Having a vaccine that induces immunity against PPR, SGP and CCPP in a single shot will offer considerable help to veterinary authorities in improving vaccine coverage in countries with dense small-ruminant populations. The multi-valent approach will maximize disease coverage through distribution networks operating effective cold chains [6,15]. Cost-effective and easily delivered vaccines decrease livestock-raising costs and improve small-ruminant trading, especially from endemic countries; indirectly, this will also improve food security by reducing mortalities resulting from such illnesses [6,18,20]. In addition, the present study used a patented liquid stabilizer that was found previously to improve vaccine delivery and stability [21].

Limitations: This study was designed as a field evaluation of the safety and immunogenicity of JOVAC-Combo^®^ and was not powered to assess protective efficacy; no virulent challenge was performed and no natural-outbreak incidence data were collected. Seroconversion was used as an indicator of vaccine-induced humoral immunity, but field-validated correlates of protection for PPR, SGP, and CCPP were not established in this study, and seropositivity should not be equated with protection against infection or clinical disease. Field staff and laboratory personnel were blinded to group allocation, and the relatively broad age range of the enrolled goats (4–9 months) may have contributed to variability in immune responses.

## Data Availability

The data presented in this study are available on reasonable request from the corresponding author.
